# FUN14 Domain Containing 1 (FUNDC1): A Promising Mitophagy Receptor Regulating Mitochondrial Homeostasis in Cardiovascular Diseases

**DOI:** 10.3389/fphar.2022.887045

**Published:** 2022-05-13

**Authors:** Yu Mao, Jun Ren, Lifang Yang

**Affiliations:** ^1^ Department of Cardiovascular Surgery, Xijing Hospital, Air Force Medical University, Xi’an, China; ^2^ Department of Cardiology and Shanghai Institute of Cardiovascular Diseases, Zhongshan Hospital Fudan University, Shanghai, China; ^3^ Department of Laboratory Medicine and Pathology, University of Washington, Seattle, WA, United States; ^4^ Department of Anesthesiology, Xi’an Children’s Hospital, Xi’an, China

**Keywords:** FUNDC1, receptor protein, mitophagy, myocardial cells, cardiovascular diseases

## Abstract

Mitochondria, the intracellular organelles for cellular aerobic respiration and energy production, play an important role in the regulation of cell metabolism and cell fate. Mitophagy, a selective form of autophagy, maintains dynamic homeostasis of cells through targeting long-lived or defective mitochondria for timely clearance and recycling. Dysfunction in mitophagy is involved in the molecular mechanism responsible for the onset and development of human diseases. FUN14 domain containing 1 (FUNDC1) is a mitochondrial receptor located in the outer mitochondria membrane (OMM) to govern mitophagy process. Emerging evidence has demonstrated that levels and phosphorylation states of FUNDC1 are closely related to the occurrence, progression and prognosis of cardiovascular diseases, indicating a novel role for this mitophagy receptor in the regulation of mitochondrial homeostasis in cardiovascular system. Here we review mitophagy mediated by FUNDC1 in mitochondria and its role in various forms of cardiovascular diseases.

## 1 Background

According to the epidemiological data from the Global Burden of Disease Study 2017 (GBD 2017), the incidence of cardiovascular disease (CVD) was increased by 21.1% from 2007 to 2017, making CVD the most prevalent cause of mortality globally, Particularly, ischemic heart disease (IHD) was increased by 22.3% and jumped atop for the years of life lost (YLLs) among all disease entities in the world (GBD 2017 Causes of Death, 2018). As an important intracellular organelle responsible for adenosine triphosphate (ATP) generation and energy metabolism, mitochondria are essential for cardiovascular homeostasis. Loss of mitochondrial integrity and function is deemed a pathological factor for alterations in cardiac structures and function (Ren et al., 2010). Mitophagy is a process to selectively remove dysfunctional or non-essential mitochondria through autophagy process, with a major role in the maintenance of cell homeostasis (Springer and Macleod, 2016). Ample experimental results have shown that damaged proteins and organelles may be degraded by autophagy in cardiomyocytes to generate amino acids and lipids, aiming to alleviate energy crisis and maintain functionality of ischemia cells (Dong et al., 2010; Wallace et al., 2010). Several mitophagy-associated receptors including NIX, BNIP3, Fun14-domain protein 1 (FUNDC1), TAX1BP1 and NDP52 participate in the activation of mitophagy (Sandoval et al., 2008; Hanna et al., 2012). In particular, FUNDC1, a novel mitochondrial receptor protein in mammalian cells, has been noted to control mitophagy stress conditions including hypoxia. A role for FUNDC1 in CVD has generated much recent attentions, in particular in myocardial ischemia/reperfusion, heart failure, septic cardiomyopathy, metabolic syndrome and other common CVD (Liu et al., 2012). In this review, the role of FUNDC1-mediated mitophagy in the pathophysiology of CVDs will be reviewed.

## 2 FUNDC1 and Mitophagy

### 2.1 Structure of FUNDC1

FUNDC1 is an outer mitochondrial membrane (OMM) protein consisting of 155 amino acid residues, where the LIR motif binds to LC3 to initiate mitophagy. FUNDC1 contains three α-helical transmembrane domains, in which the C-terminal region resides in the intermembrane and the N-terminal region faces the cytoplasm with a segment of LIR. As aforementioned, other than FUNDC1, several mitophagy receptors including NIX and BNIP3 also contain LIR motifs. Although LIR motifs of these mitophagy receptors are distinct from each other, they all possess the common structural formula: a-xx-b, where “a” represents an aromatic amino acid (W/F/Y), “b” denotes an aliphatic amino acid (L/I/V), and “x” can be any amino acid (Birgisdottir et al., 2013). The specific structure of LIR for FUNDC1 is Y18-E19-V20-L21, which docks with Y and L pockets of LC3 through hydrophobic action and triggers initiation of mitophagy (Kuang et al., 2016). In this context, FUNDC1-mediated mitophagy is highly dependent upon direct interaction between its LIR motifs and LC3 (Gustafsson and Dorn, 2019).

### 2.2 FUNDC1-Mediated Mitophagy

**FIGURE 1:**
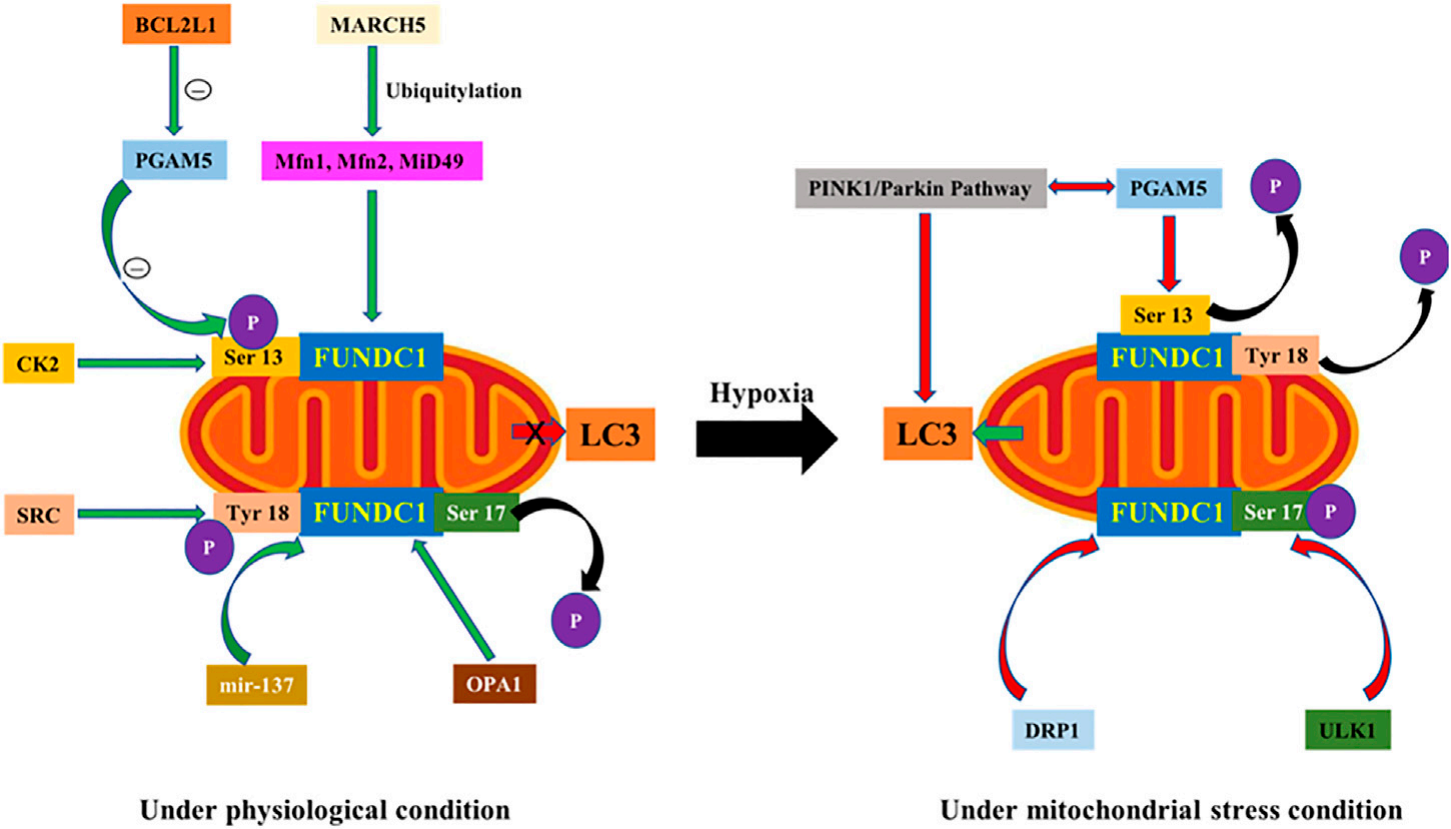
**(A)**. Under physiological condition, 1) Ser^13^ in FUNDC1 is phosphorylated by CK2 kinase and Tyr^18^ by SRC kinase, and their phosphorylation interferes with FUNDC1 interaction with LC3 motif to inhibit mitophagy. 2) BCL2L1 interacts with PGAM5 through BH3 domain to inhibit PGAM5 activation thus suppressing Ser^13^ dephosphorylation. 3) Aggregated MARCH5 is separated and interacted with FUNDC1, which degrades FUNDC1 through Parkin-mediated ubiquitination to dampen binding of FUNDC1 with LC3. 4) OPA1 interacts with FUNDC1 through Lys70 residue. **(B)**. Under mitochondrial stress, 1) ULK1 activates Ser^17^ to upregulate FUNDC1 phosphorylation. 2) PGAM5 is the phosphatase of Ser^13^, which turns to dephosphorylate Ser^13^ under hypoxic setting. PGAM5-FUNDC1 cascade plays a synergistic role with PINK1-Parkin axis in mitophagy. 3) mir-137 inhibits mitophagy by downregulating FUNDC1 under hypoxia. 4) Dephosphorylation of FUNDC1 promotes dissociation of FUNDC1-OPA1 complex, leading to the binding of DRP1 to recruit DRP1 onto mitochondrial membrane to evoke fission.

Under physiological condition, FUNDC1 exists in phosphorylated form with little mitophagy activity. FUNDC1-mediated mitophagy is mainly evoked in response to ischemia, hypoxia and mitochondrial membrane potential collapse. To-date, several main regulatory machineries have been identified including casein kinase 2 (CK2), SRC kinase, PGAM5 phosphatase, MARCH5 and microRNA-137 (Wu et al., 2016). Phosphorylation and dephosphorylation of Ser^17^, Ser^13^ and Tyr^18^ in FUNDC1 LIR determine how tightly FUNDC1 conjugates with LC3. When mitophagy is supposedly triggered by external stimuli, Ser^17^ is phosphorylated while Ser^13^ and Tyr^18^ are dephosphorylated, which form hydrogen bonds with Lys^49^, Arg^10^ and Asp^19^ side chains in LC3 motif, respectively (Kuang et al., 2016; Lv et al., 2017). Therefore, it is pertinent to elucidate the regulatory mechanisms of phosphorylation of Ser^17^, Ser^13^ and Tyr^18^ for an overachieving picture of FUNDC1-mediated mitophagy (Figure 1). Currently, regulators of FUNDC1 phosphorylation mainly include CK2, SRC and ULK1, while PINK1, PGAM5 and BCL2L1 are deemed key regulators of FUNDC1 dephosphorylation.

#### 2.2.1 Phosphorylation Regulation of FUNDC1

FUNDC1-mediated mitophagy is regulated by reversible phosphorylation. Under physiological condition, Ser^13^ in FUNDC1 is phosphorylated by CK2 kinase and Tyr^18^ is phosphorylated by SRC kinase, and their high phosphorylation status inhibits their interaction with LC3 to prevent mitophagy. Under the stimulation of oxygen deficiency or carbonyl cyanide 4-(trifluorome-thoxy) phenylhy-drazone (FCCP), dephosphorylation of Ser^13^ and Tyr^18^ enhanced the interaction between FUNDC1 and LC3, triggering mitophagy (Liu et al., 2014). Therefore, phosphorylation of Tyr^18^ is deemed a molecular switch for FUNDC1-mediated mitophagy (Kuang et al., 2016). In addition, concerted studies using ULK1 defective mutants with FUNDC1 knockout model suggest that FUNDC1 is the mitochondrial localization substrate of ULK1 and may have a ULK1 adapter property (Wu et al., 2014a). Further studies showed that SRC kinase inhibited the binding of ULK1 to mitochondria and decreased FUNDC1 phosphorylation of ULK1 on Ser^17^. Thus, ULK1 and FUNDC1 are both necessary for mitophagy, and regulate mitophagy synergistically, in contrary to inhibition of mitophagy through phosphorylation of Ser^13^ and Tyr^18^. Therefore, phosphorylation of Ser^13^, Ser^17^, and Tyr^18^ on FUNDC1 residues influences their interaction with LC3 together, thereby promoting or inhibiting mitophagy.

#### 2.2.2 Dephosphorylation Regulation of FUNDC1

It is well known that collapse of mitochondrial membrane potential causes the accumulation of PINK1, which recruits Parkin onto damaged mitochondria to turn on mitophagy (Narendra et al., 2010). Studies proposed that the receptor-mediated machinery is involved in mitophagy induction by a decline in mitochondrial membrane potential (Chen et al., 2014). PGAM5, the phosphatase of Ser^13^, dephosphorylates Ser^13^ under hypoxia, and plays a synergistic role with PINK1-Parkin pathway in mitophagy. It was observed that PINK1 interacts with PGAM5, and deficiency of PGAM5 inhibits PINK1-mediated mitophagy (Imai et al., 2010). Knockout of FUNDC1 reduces Parkin translocation to mitochondria (Chen et al., 2014). Notably, PGAM5 activation is closely related to the anti-apoptotic protein BCL2L1. Wu and others found that BCL2L1 interacts with PGAM5 through BH3 domain to inhibit PGAM5 activation and prevents Ser^13^ dephosphorylation under normal condition. When hypoxia occurs, BCL2L1 degrades and releases PGAM5 to prompt Ser^13^ dephosphorylation, resulting in the initiation of FUNDC1-mediated mitophagy (Wu et al., 2014b). Levels of BCL2L1 govern FUNDC1 dephosphorylation and mitophagy mediated by PGAM5 through gain- and loss-of- function studies. Knockdown of PGAM5 inhibited mitophagy regardless of BCL2L1 level. Therefore, the BCL2L1-PGAM5-FUNDC1 signaling axis is vital for mitophagy under hypoxic condition.

#### 2.2.3 Non-phosphorylation-dependent Negative Regulation of FUNDC1

MARCH5, a E3 ubiquitin ligase in OMM, participates in the regulation of mitochondria through ubiquitination of mitochondrial fusion protein 1 (Mfn1), mitochondrial fusion protein 1 (Mfn2), and MiD49 (Sugiura et al., 2013; Park et al., 2014; Xu et al., 2016). Recently, MARCH5 was also shown to participate in FUNDC1-mediated mitophagy. Under hypoxia, aggregated MARCH5 interacted with FUNDC1, which degrades FUNDC1 through Parkin-mediated ubiquitination and results in reduced binding of FUNDC1 with the LC3 motif, and desensitization of mitochondria (mitophagy) to hypoxia (Chen et al., 2017). These results suggest that MARCH5 may negatively regulate FUNDC1-mediated mitophagy, to maintain mitophagy at a certain level to avoid excessive autophagy damage of intracellular homeostasis. In addition, microRNA-137 (mir-137) may also inhibit mitophagy by down-regulating FUNDC1 under hypoxia both *in vivo* and *vitro* (Li et al., 2014). Interestingly, overexpression of mir-137 inhibited mitophagy without influencing autophagy of other organelles and proteins. These results indicate that mir-137 specifically regulates FUNDC1-mediated mitophagy, revealing promises for drug development targeting FUNDC1-mediated mitophagy.

#### 2.2.4 FUNDC1 and Mitochondrial Dynamics

Mitochondria are highly dynamic organelles, with their division, fusion and selective autophagy governing morphology and quality of mitochondria. Mitochondrial dynamics refers to a process in which mitochondria maintain dynamic balance through fusion and fission, and imbalance between the two is usually the prerequisite for mitophagy (Chen et al., 2016). After mitochondrial division, polarized and depolarized mitochondria are generated. The polarized mitochondria could be refused, while the depolarized daughter mitochondria become the target of mitophagy and could be cleared away (Mattenberger et al., 2003; Twig et al., 2008). Mitochondrial fusion is mainly mediated by Mfn1 and Mfn2 in OMM and optic atrophy 1 (OPA1) in the inner mitochondrial membrane (IMM). On the other hand, mitochondrial fission is mainly mediated by dynamic related protein 1 (DRP1) (Ding and Yin, 2012). OPA1 interacts with FUNDC1 through Lys70 residue. When Lys70 is mutated, the interaction of OPA1 and FUNDC1 is disengaged to promote mitophagy. Under mitochondrial stress, dephosphorylation of FUNDC1 promotes the dissociation of FUNDC1-OPA1 complex to bind with DRP1 to recruit DRP1 in mitochondria. In addition, Wu and coworkers noted division of mitochondria prior to the engulfment by autophagic pro-body (Wu et al., 2016). FUNDC1 aggregates onto the mitochondrial membrane to interact with endoplasmic reticulum (ER) calcium-binding protein (CANX) under hypoxia. The N-terminus of CANX binds to the hydrophilic region of FUNDC1, an indirect process due to the localization of N-terminus of CANX within the ER lumen. Therefore, contribution from intermediate proteins is speculated between FUNDC1 and CANX. As mitophagy proceeds, FUNDC1 would dissociate from CANX and recruit DRP1 to drive mitochondrial fission in response to hypoxic stress. Compared to CANX, DNM1L also binds to the FUNDC1 hydrophilic zone. In addition, deletion of FUNDC1, DRP1 or CANX in hypoxic cells may provoke mitochondrial elongation, elevated number of elongated mitochondria, decreased co-location of autophagosomes and mitochondria, thus preventing mitophagy. FUNDC1 is a novel receptor for DRP1 and may be an important molecule in the regulation of mitochondrial fission and autophagy under hypoxia (Wu et al., 2016). These results suggest that FUNDC1 regulates mitochondrial fission/fusion and mitophagy by interacting with DRP1 and OPA1, and functions as a springboard between mitochondrial dynamics and mitophagy.

## 3 Role of FUNDC1 in Pathogenesis of Various CVDs

Mitophagy plays a rather important role in the maintenance of cell homeostasis. On one hand, mitophagy helps to maintain the stability of intracellular homeostasis. On the other hand, defective mitophagy predisposes pathological processes. Recent findings have depicted a key role for mitophagy in the pathogenesis of a variety of CVDs (Dong et al., 2010). Given by regulating apoptosis, oxidative stress and calcium homeostasis and biological energy, mitochondria is the main regulatory factor of stress reaction corresponding to various types of CVDs. Nonetheless, the precise role of how FUNDC-mediated mitophagy contributes to pathogenesis of cardiovascular diseases remains elusive.

### 3.1 FUNDC1 and Myocardial Ischemia/Reperfusion

**FIGURE 2:**
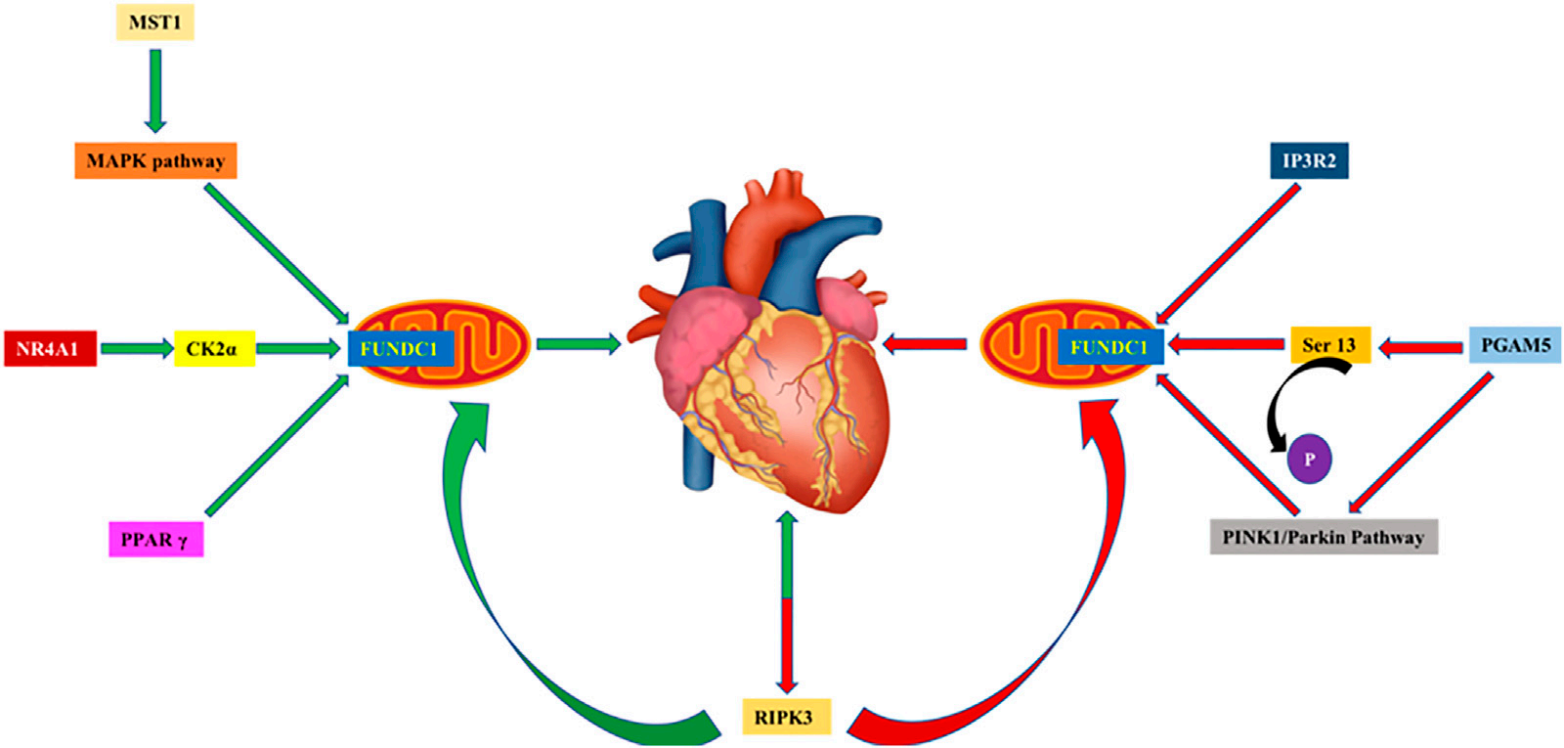
1) By interacting with IP3R2, knocked-out FUNDC1 in cardiomyocytes compromises mitochondrial function, disrupts MAMs and Ca^2+^ influx into mitochondria and cytosols. 2) PGAM5 dephosphorylates FUNDC1 at Ser^13^ and activates mitophagy through binding with LC3 motif on phagocytes. PGAM5 provides cytoprotection against necroptosis by promoting PINK1-mediated mitophagy, and PGAM5 injury aggravates necroptosis caused by I/R injury in hearts and brains. 3) Upregulation of MST1 inhibits FUNDC1-mediated mitophagy through MAPK in I/R injury, increases ROS production and promotes cell apoptosis. 4) NR4A1 activates CK2α during MI/R, leading to the inhibition of FUNDC1-mediated mitophagy and aggravation of I/R injury. 5) PPAR γ activates FUNDC1-mediated mitophagy, enhances mitochondrial function and ATP generation. 6) Reperfusion injury disrupts FUNDC1-mediated mitophagy and triggers caspase 9-related apoptosis through upregulation of RIPK3. RIPK3 deficiency protects against I/R injury through activation of mitophagy and inhibition of apoptosis.

Coronary blockade evoked by thrombosis may lead to myocardial ischemia injury, resulting in angina pectoris, myocardial infarction, sudden cardiac death and other symptoms. However, restoration of blood supplement after ischemia could trigger secondary myocardial injury–namely myocardial ischemia/reperfusion (MI/R) injury due to ROS production, Ca^2+^ overload, neutrophils aggregation and inflammation. Platelet activation and microvascular injury are upstream events for myocardial cell injury and pathological MI/R injury (Zhou et al., 2017a). Studies in the past few decades have focused on the downstream mechanisms of MI/R tolerance. In recent years, the influence of platelet activation and microvascular disorders on myocardial cell injury after MI/R has gradually attracted our attention, offering a new treatment option for myocardial injury. FUNDC1 is closely associated with mitophagy regulation in platelets, microvascular endothelial cells and cardiomyocytes under MI/R insult. Currently, researchers have ascribed a beneficial role of FUNDC1-mediated mitophagy in the protection against cardiac MI/R injury. It not only explains that mitosis associated with FUNDC1 phagocytosis abates the sequence of events (Zhou et al., 2017a), but also provides a promising therapeutic tool for the treatment of acute cardiac injury (Figure 2).

#### 3.1.1 Fine-Tune of FUNDC1-Mediated Mitophagy in Myocardial Protection

Cardiomyocytes are rich in mitochondria for energy production, in order to maintain normal systolic and diastolic function of the heart (Barth et al., 1992). If mitochondria are injured, cardiomyocyte function will be compromised to trigger senescence of cardiomyocytes (Marzetti et al., 2013; Ikeda et al., 2014). In addition, severe damage of mitochondria may also release pro-apoptotic factors, leading to programmed cell death and myocardial injury (Zhou et al., 2018a). Injured cardiomyocytes could stimulate mitophagy to remove long-lived or damaged mitochondria, for the maintenance of cellular homeostasis and myocardial function (Kubli et al., 2013; Feng et al., 2016). When H9c2 cells are subjected to hypoxic challenge, WD repeat domain 26 (WDR26), a protein evoked by ischemic preconditioning, translocates Parkin to mitochondria by increasing mitochondrial membrane potential, thus promoting mitochondrial protein ubiquitination, mitophagy and myocardial protection (Feng et al., 2016). However, oxidative stress leads to excess mitophagy, which results in the degradation of healthy mitochondria despite the initial goal of selective clearance of damaged mitochondria. Unchecked mitophagy often causes irreversible damage to cardiomyocytes. Jin and team depicted that dual specificity protein phosphatase1 (DUSP1) was capable of inhibiting BNIP3-mediated mitophagy through a JNK-mediated mechanism, enhancing cardiomyocyte function following MI/R (Jin et al., 2018). These studies further consolidated an important role for mitophagy (FUNDC1-dependent or -independent) in cardiovascular system, suggesting the utility of appropriately enhanced mitophagy or inhibition of excessive mitophagy in the prevention of myocardial injury under pathological condition.

#### 3.1.2 Regulation of FUNDC1 With Platelet Activation

The main function of platelets is to participate in hemostasis and coagulation, in order to protect against vascular endothelial damage and atherosclerosis from external stimuli or endogenous reactions. However, platelets are over-activated by coagulation factors, ADP and inflammatory cells during MI/R. Activated platelets are spontaneously aggregated to form microthrombus, to block microvasculature and trigger myocardial cell injury or MI (Randriamboavonjy et al., 2015; Zhang et al., 2017). Platelet activation evokes dramatic energy-demanding morphological changes. Thus, maintenance of energy balance and mitochondrial integrity is essential to platelet activation and aggregation (Chen et al., 2016; Zhang et al., 2017). On one hand, mitophagy may avert further activation of platelets and formation of thrombosis by removing damaged mitochondria in platelets, offering a protective role in diabetic patients (Lee et al., 2016). On the other hand, insufficient mitophagy causes mitochondrial dysfunction of platelet and poor ATP production, leading to dissimilation of platelet activation and aggregation (Jang et al., 2015). It was reported that mitophagy may be facilitated through post-transcriptional modification of FUNDC1 in platelets, as evidenced by the absence of mitophagy activity in mice with knockout of platelets (Zhang et al., 2016). In addition, Zhang and others proposed that platelet mitophagy plays a dual role in the regulation of platelet activity and MI/R injury (Zhang et al., 2016). In the early stage of hypoxia, FUNDC1 binds with LC3 to induce mitophagy, which removes long-lived or damaged mitochondria to sustain mitochondrial function, in conjunction with accelerated mitochondrial metabolism and ATP production during initial myocardial repair. In addition, with prolonged ischemia, mitophagy is overactivated to prevent further deterioration of myocardial injury at the expense of removing parts of normal mitochondria (Zhang et al., 2016). These results suggest that the heart may execute a self-protective machinery through mitophagy induction in response to adverse external stimuli. These studies suggest a vital role for FUNDC1-mediated mitophagy in platelet activation and ischemia-reperfusion injury. During the ischemia period, FUNDC1-mediated mitophagy was upregulated in platelets and cardiomyocytes in response to ischemia and hypoxia challenge (Zhang et al., 2016).

#### 3.1.3 Phosphorylation of FUNDC1 With Ser^13^, Ser^17^ and Tyr^18^ in MI/R

FUNDC1 acts as a mitophagy receptor under hypoxia, through phosphorylation of Ser^13^, Ser^17^ and Tyr^18^. By interaction with IP3R2 (inositol 1, 4, 5-phosphoinositol receptor), FUNDC1 knockout in cardiomyocytes compromises mitochondrial function and disrupts mitochondrial-associated membranes (MAMs) and Ca^2+^ influx into mitochondria and cytosols (Wu et al., 2017). PGAM5 dephosphorylates FUNDC1 at Ser^13^ and activates mitophagy through binding to LC3 motif on phagocytes (Chen et al., 2014). PGAM5 also provides cytoprotection against necroptosis by promoting PINK1-mediated mitophagy, and PGAM5 injury aggravates necroptosis caused by MI/R injury in both hearts and brains (Lu et al., 2016). In addition, phosphorylation of FUNDC1 at Tyr^18^ remains virtually unchanged during post-MI/R phase, whereas phosphorylation of FUNDC1 at Ser^13^ is progressively upregulated, suggesting that inactivation of FUNDC1 through Ser^13^ following reperfusion exposure. It is important to realize that levels of phosphorylated FUNDC1 at Tyr^18^ and Ser^13^ are downregulated during ischemia prior to a progressive upregulation during refusion. Thus, inhibition of FUNDC1-mediated mitotic phagocytosis is a pathologic cause of MI/R injury, and phosphorylation of FUNDC1 at Ser^13^ and Tyr^18^ is closely associated with myocardial injury, indicating their diagnostic and prognostic potential as biomarkers for acute MI.

#### 3.1.4. Regulation by MST1, CK2α, RIPK3 and PPAR γ in MI/R

Dysfunctional mitochondria are closely related to the occurrence and progression of various CVDs, amongst these findings play a critical role. Upregulation of mammalian STE20-like kinase 1 (MST1) inhibits FUNDC1-mediated mitophagy through the cAMP response element binding protein (MAPK/ERK-CREB) cascade in MI/R injury, leading to ROS production, cell apoptosis, and aggravation of myocardial injury. Ablation of MST1 gene preserves FUNDC1 levels and mitophagy, thus reducing myocardial infarction size and preserving cardiac function (Yu et al., 2019). It was reported that casein kinase 2α (CK2α) levels were elevated in MI/R injury, which inhibited FUNDC1-mediated mitophagy. Cardiac-specific deletion of CK2α was reported to rescue against mitochondrial damage and protect cardiac function by reversing mitophagy in a FUNDC1-dependent manner (Zhou et al., 2018a). In addition, NR4A1 activates CK2α during MI/R, leading to suppressed FUNDC1-mediated mitophagy and aggravated MI/R injury (Zhou et al., 2018b). Similarly, reperfusion injury disrupted FUNDC1-mediated mitophagy and led to caspase 9-related apoptosis through upregulation of receptor-interacting serine/threonine kinase 3 (RIPK3). RIPK3 deficiency protected against MI/R injury by activation of mitophagy and inhibition of apoptotic pathways (Zhou et al., 2017b). Zhou and others reported that loss of peroxisome proliferator-activated receptor γ (PPAR γ) activates FUNDC1-mediated mitophagy and enhances mitochondrial function and ATP generation, leading to platelet aggregation and cardiac dysfunction in MI/R injury (Zhou et al., 2017a).

### 3.2 FUNDC1 and Heart Failure

Heart failure is associated with high mobility and mortality rate worldwide. Structural cardiac remodeling, including myocardial hypertrophy and fibrosis, was shown to significantly affect ventricular dysfunction in patients with heart failure (Shen et al., 2015). In addition, abnormal mitochondrial metabolism is an important pathological basis of heart failure. To understand the role of mitochondrial metabolism in the development of heart failure is helpful for early detection of risk factors and application of effective treatment to improve the prognosis of heart failure. As described below, mitochondrial metabolism is under the close scrutiny of a number of cellular signal mechanisms including aldehyde dehydrogenase 2 (ALDH2), Alpha-lipoic acid (α-LA) and IP3R2. Their indispensable roles in the pathogenesis of heart failure reveal that targets above might be a potential therapeutic option for heart failure and other CVDs (Sun et al., 2014; Shen et al., 2017).

#### 3.2.1 Regulation of α-LA and ALDH2 for FUNDC1-Mediated Mitophagy

Aldehyde dehydrogenase 2 (ALDH2) is the most active isoenzyme in the ALDH superfamily, which mainly resides in mitochondria. ALDH2 possesses three main enzymatic functions, including dehydrogenase, esterase and reductase, among which dehydrogenase function (in removing aldehyde metabolites) is the most well-studied. Studies demonstrated that important cardioprotective effects in various risk factors of heart failure, such as coronary artery disease (CAD), hypertension, diabetes, alcoholism and other susceptibilities (Sun et al., 2014; Shen et al., 2015; Wang et al., 2016; Shen et al., 2017). Alpha-lipoic acid (α-LA) is a coenzyme in mitochondria. It is a cofactor of pyruvate dehydrogenase complex, ketoglutarate and amino acid hydrogenase complex which belongs to B vitamins. Studies have shown that α-LA restores ALDH2 activity through reduction of disulfide at its active site in diabetic cardiomyopathy and acute MI/R injury, thus restoring ALDH2 activity and improving nitrate tolerance (Wang et al., 2011; He et al., 2012). In the transverse aortic contraction (TAC) model, α-LA was shown to reduce cardiac hypertrophy (Zhang et al., 2014). As a highly efficient metabolic enzyme in mitochondria, increasing evidence supports an important role of ALDH2 in heart failure (Marino and Levi, 2018). Increased levels or activity of ALDH2 provides cardiac protection of ischemia and atherosclerosis through aldehyde detoxification (Chen et al., 2008; Yang et al., 2018). Li and others found that reduced α-LA in TAC-induced cardiac hypertrophy and cardiac fibrosis in an ALDH2-dependent manner. Mechanistically, α-LA activates FUNDC1 through NRF1 signaling (Li et al., 2020). However, it is not clear whether ALDH2 regulates FUNDC1 or other forms of mitophagy under pathological pressure overload settings. FUNDC1 mitophagy was found downregulated in pressure-overload induced heart failure, the effect of which was restored by α-LA treatment, in an ALDH2 dependent manner (Li et al., 2020). From the microarray data, certain mitochondria and autophagy related genes, such as Atpif1, BECN2 and BNIP3 were robustly altered, suggesting a role for these genes in α-LA- and ALDH2-offered action (Li et al., 2020).

#### 3.2.2 Role of IP3R2 in FUNDC1-Regulated Heart Failure

Recent studies confirmed that FUNDC1 promotes mitochondria-associated ER membranes (MAMs) stability and regulates ER release of Ca^2+^ ions into mitochondria and cytoplasm through interaction with IP3R2 (Wu et al., 2017). MAMs refer to membranous contact of mitochondrial ER connection, with an important role in maintaining Ca^2+^ homeostasis (Rowland and Voeltz, 2012; Giorgi et al., 2015). FUNDC1 regulates the formation and maintenance of MAMs, and the destruction of FUNDC1-IP3R2 axis on MAMs promotes development of heart failure (Wu et al., 2017). Knockout of FUNDC1 in murine cardiomyocytes leads to more abundant slender mitochondria, and accumulation of defective mitochondria with suppressed cardiac function. Echocardiography showed cardiac systolic and diastolic dysfunction, myocardial fibrosis and increased expression with stress genes, including cardiac natriuretic peptide and brain natriuretic peptide, in FUNDC1 knockout mice. When subjected to acute myocardial infarction (AMI), FUNDC1 knocked-out mice exhibited worsened heart failure, shortened survival and greater mortality. Further studies suggested that FUNDC1 knockout exacerbated AMI-induced heart failure by inhibiting MAMs formation. This supports the idea that FUNDC1 and MAMs are involved in the development of heart failure (Giorgi et al., 2015). Therefore, restoration of MAMs integrity may be a new therapeutic target for heart failure.

### 3.3 FUNDC1 and Septic Cardiomyopathy

The incidence of septic cardiomyopathy ranges from 18% to 29% in septic patients with a high mortality rate and poor treatment option (Tan et al., 2019). Sepsis cardiomyopathy is characterized by left ventricular diastolic dysfunction, and severe sepsis syndrome leads to impaired ejection fraction (Hollenberg and Singer, 2021). Oxidative stress, cytokine overproduction, microvascular injury, and ATP metabolism of cardiomyocytes have been reported as potential molecular mechanisms of sepsis-induced myocardial injury (Tan et al., 2021; Vasques-N´ovoa et al., 2020; Sanfilippo et al., 2019; Zhong et al., 2019; Honda et al., 2019; Martin et al., 2019). Mitochondrial dysfunction plays an important role in inducing oxidative stress and promoting energy crisis (Tan et al., 2019). These changes are always accompanied by apoptosis or necroptosis of myocardial cells (Kokkinaki et al., 2019; Wang et al., 2020a). Irreversible cardiomyocyte death is a key molecular mechanism that activates inflammatory responses. The pathophysiology of septic cardiomyopathy was reported that mainly related to the following mechanisms: cardiac toxicity factors [such as tumor necrosis factor-α, C-reactive peptide (CRP), interleukin-6, complement and endotoxin], inflammation caused by excessive production, catecholamine toxicity caused by sympathetic activation (Ehrman et al., 2018). The molecular mechanisms associated with septic myocarditis include oxidative stress, calcium overload, ATP deficiency, autophagy inactivation, metabolic reprogramming, mitochondrial dysfunction, ER stress and induction of apoptosis and necroptosis (Ehrman et al., 2018; Tan et al., 2019). Therein, mitophagy and the mitochondrial unfolded protein response (UPRmt) are the predominant stress-responsive and protective mechanisms involved in repairing damaged mitochondria.

#### 3.3.1 Role of UPR^mt^ in Mitophagy

In order to maintain a functional mitochondrial network, mitochondria develop specific repair pathways, including mitochondrial bacteriophage and UPR^mt^ (Zhu et al., 2021a; Gottlieb et al., 2021). UPR^mt^ prevents abnormal protein accumulation in mitochondria by normalizing folding and degradation of mitochondrial protein (Shpilka et al., 2021). UPR^mt^ controls the dynamic import and export of mitochondrial proteins and fine-tunes mitochondrial behavior (Lim et al., 2021). Recently, a role of UPR^mt^ in myocardial stress was reported (Smyrnias et al., 2019; Wang et al., 2019). In a mouse model of chronic stress-mediated cardiac hypertrophy (Smyrnias et al., 2019), UPR^mt^ markers such as ATF5, CHOP, mtDNAj, ClpP and LonP1 were significantly elevated at the mRNA level, suggesting a role for UPR^mt^ as an adaptive responsive molecule to myocardial stress. Interestingly, further activation of UPR^mt^ by nicotinamide nucleoside supplementation was associated with normal mitochondrial respiration and reduced cardiomyocytes *in vitro* (Smyrnias et al., 2019). In addition, cardiomyocyte apoptosis was lessened and cardiac function was improved in response to nicotinamide nucleoside, suggesting a cardioprotective effect activated by UPR^mt^ (Smyrnias et al., 2019). Loss of FUNDC1 significantly upregulated the transcription of UPR^mt^ markers, indicating that it acts as a compensatory mechanism in response to mitophagy inactivation. Wang and others reported that activation of FUNDC1 mitophagy alleviated mitochondrial injury and cardiomyocyte death in a cell model of septic cardiomyopathy (Wang et al., 2021). Increased mitochondrial quality control activates UPR^mt^ to optimize the import and export of mitochondrial proteins by upregulating gene transcription or promoting protein degradation (Wang et al., 2020b; Wang and Zhou, 2020). If UPR^mt^ cannot repair mitochondrial damage, mitochondrial division is induced to segregate the damaged regions from the healthy mitochondrial network (Zhou et al., 2018c; Wang et al., 2020c).

#### 3.3.2 Role of Mitotic Phagocytosis

Mitophagy is used as a scavenger to remove structurally damaged mitochondria (Zhou et al., 2017b; Zhou et al., 2018a). Due to mitophagy-mediated mitochondrial removal is usually accompanied by reduction of mitochondria, mitochondrial integrity is enhanced by mitophagy to jack up ATP synthesis (Zhou and Toan, 2020; Chang et al., 2021). Therefore, UPR^mt^ and mitotic phagocytosis can be considered as distinct mitochondrial repair pathways. The former controls mitochondrial proteomics, while the latter alters mitochondrial quantity. Both regulatory and pathophysiological roles of mitotic phagocytosis were identified in vascular diseases (Zhu et al., 2021b; Zhou et al., 2021). Moderate activation of mitotic phagocytosis alleviates myocardial stress, while induction of abnormal mitotic phagocytosis is unexpectedly associated with pronounced cardiomyocyte death due to abrupt drops in residual mitochondria and intracellular ATP. Activation of FUNDC1-associated mitotic phagocytosis was shown to protect the heart against lipopolysaccharides (LPS)-induced sepsis through preserving mitochondrial function and structure (Wang et al., 2021). Moreover, loss of FUNDC1 inhibits mitotic phagocytosis, resulting in mitochondrial dysfunction and myocardial cell death. This finding further sheds some lights on the role of FUNDC1-dependent mitotic phagocytosis in myocardial stress (Zhou and Toan, 2020). Based on this finding, targeting FUNDC1-dependent mitotic phagocytosis should be a potential strategy for reconciliation of cardiac dysfunction in sepsis.

### 3.4 Expression of FUNDC1 in Metabolic Heart Diseases

Metabolic syndrome (MetS) is a group of complex metabolic disorders, which refers to a group of cardiovascular risk factors, including insulin resistance, obesity, dyslipidemia, glucose intolerance and elevated blood pressure (Li et al., 2021a). Metabolic syndrome caused by high fat and high calorie intake as well as satiety is becoming a global epidemic and a major health concern (Saklayen, 2018). Disturbances in cardiac glucose and lipid metabolism inevitably lead to abnormalities in energy production and oxygen usage. In particular, increasing evidence noted that mitochondrial dysfunction is a cardinal event in diabetic or obesity cardiomyopathy (Brownlee, 2001; Green and Kroemer, 2004).

#### 3.4.1 Main Mechanisms of Mitophagy in MetS

In MetS, glucose and lipid metabolism can be improved by reducing oxidative damage and promoting mitochondrial networking (Kelley et al., 2002). Under hypoxia, reducing mitochondrial number prevents production of reactive oxygen species (ROS) evoked by the mismatch between oxygen consumption and mitochondrial abundance (Lowell and Shulman, 2005; Giorgi et al., 2009). In addition, metabolic stress-mediated vascular endothelial injury can be prevented by promoting mitophagy in obese and diabetic situation (Vance et al., 1997). Tong and associates demonstrated that preservation or activation of mitophagy may prevent HFD-induced diabetic cardiomyopathy (Tong et al., 2019). Likewise, FUNDC1 deficiency was shown to promote obesity, insulin resistance, MetS, cardiac remodeling, and even cell death due to defective control of mitochondrial quality (Wu et al., 2019a; Ren et al., 2020). These findings suggest a central role for FUNDC1 in the regulation of cardiac function in obese MetS and the role of FUNDC1-mediated mitophagy as a main regulator of mitochondrial function, particularly in response to hypoxia-mediated mitochondrial injury commonly found in metabolic diseases.

#### 3.4.2 Regulation of Ca^2+^, IP3R2 and IP3R3 With FUNDC1

Studies has revealed that high glucose-induced AMPK inhibition contributes to the onset of diabetic cardiomyopathy by upregulation of FUNDC1, FUNDC1-governed MAMs, and rises in mitochondrial Ca^2+^ in the heart. Mitochondrial Ca^2+^ controls cell metabolism and cell death through necroptosis, apoptosis, and autophagy. In particular, transient fluctuations of Ca^2+^ activate three matrix dehydrogenases (pyruvate dehydrogenase, α ketoglutarate dehydrogenase, and isocitrate dehydrogenase) governing oxidative phosphorylation and dehydrogenase which stimulate ATP generation (Denton, 2009). Recent findings also suggested an essential role for FUNDC1 in maintaining MAMs structures and ensuring adequate Ca^2+^ transfer from ER to mitochondria (Wu et al., 2017). Wu and others reported that FUNDC1 interacted with IP3R2 and thus inhibited IP3R2 ubiquitination and proteasome degradation, leading to activation of high glucose-driven adenylate activated protein kinase (AMPK). Interestingly, downregulation of FUNDC1 loosened FUNDC1-governed MAMs to reverse diabetic cardiomyopathy through inhibition of mitochondrial Ca^2+^ overload and mitochondrial injury (Wu et al., 2019b). In line with this finding, results from the British Prospective Diabetes Study suggested that metformin, one common antidiabetic drugs for type 2 diabetes and AMPK activator (Zhou et al., 2001; Xie et al., 2008; Xie et al., 2011; Meng et al., 2015), improved cardiac function and reduced incidence of myocardial infarction in diabetic patients (Effect of intensive blood, 1998). In contrary to its role in diabetic hearts, deficiency of FUNDC1 was found to accentuate obesity cardiomyopathy through regulation of mitochondrial Ca^2+^ integrity in an IP3R3-depednent manner. Data from our group revealed that FUNDC1 directly interacts with subunit of human SCF ubiquitin ligase complex FBXL2 to ease degradation of IP3R3 and reduce mitochondrial Ca^2+^ overload (Ren et al., 2020). These findings suggest an apparent paradoxical role of FUNDC1 in distinct settings of metabolic heart anomalies.

## 4 Conclusion

Taken together, ample evidence has favored a unique role for FUNDC1-mediated mitophagy in the pathogenesis and management of CVD. Accumulating observations from FUNDC1 studies since its initial discovery a decade ago has undoubtedly consolidated its role as a new target for treatment of human diseases including CVDs (Li et al., 2021b; Liu et al., 2021). Although substantial progresses were achieved for the role of mitophagy in CVDs (Ajoolababy et al., 2020; Ajoolababy et al., 2022), the precise regulatory mechanisms, especially in connection with kinases and phosphorylation, remains to be explored. Recent finding from our group indicated a beneficial role for pentacyclic triterpene oleanolic acid in facilitating FUNDC1-mediated mitophagy (Gong et al., 2022), although drug development for small molecule regulator of FUNDC1 remains dismal. Moreover, moderate controllability of mitophagy is rather difficult to handle, thus making application of mitophagy inducers rather challenging. It is of great practical significance to elucidate the precise regulatory mechanism of FUNDC1 in the cardiovascular diseases at the molecular level. In-depth understanding of regulatory machineries and critical links of FUNDC1-dependent mitophagy in human diseases will provide new strategies and treatments around this important mitophagy receptor.
